# Composition Optimization and Mechanical Properties of Mg-Al-Sn-Mn Alloys by Orthogonal Design

**DOI:** 10.3390/ma11081424

**Published:** 2018-08-13

**Authors:** Maosheng Guan, Yaobo Hu, Tianxu Zheng, Tianshuo Zhao, Fusheng Pan

**Affiliations:** 1 College of Materials Science and Engineering, Chongqing University, Chongqing 400044, China; smiles94@163.com (M.G.); tianxu.zheng@cqu.edu.cn (T.Z.); tszhao@cqu.edu.cn (T.Z.); fspan@cqu.edu.cn (F.P.); 2 National Engineering Research Center for Magnesium Alloys, Chongqing 400044, China

**Keywords:** magnesium alloys, orthogonal design, grain refinement, mechanical properties

## Abstract

Nine kinds of rare-earth free Mg-Al-Sn-Mn magnesium alloys were designed by orthogonal method. Scanning electron microscopy (SEM), energy dispersive spectrometry (EDS), X-ray diffraction (XRD), electron backscatter diffraction (EBSD), and tension tests were carried out to investigate the microstructures and mechanical properties. As-cast Mg-Al-Sn-Mn alloys have an obvious dendritic structure that is composed of α-Mg, Mg_17_Al_12_, and Mg_2_Sn phases. After hot extrusion, the cast dendrite structure changed into a recrystallized equiaxed grain. Mg_17_Al_12_ dissolved completely into a matrix, and only α-Mg, Mg_2_Sn, and a few Al-Mn phases could be observed. The influence of three alloy elements (Al, Sn, and Mn) on grain size, texture intensity, ultimate tensile strength (UTS), tensile yield strength (TYS), and elongation (EL) were studied by extreme difference analysis method. The content of Mn had the greatest influence on grain size. The AT61-0.2Mn and AT73-0.2Mn alloys had the smallest grain, reaching 6.8 μm. The content of Al had the greatest influence on the strength; therefore, the AT73-0.2Mn alloy had the highest UTS, 322 MPa, and TYS, 202 MPa. The content of Sn had the greatest influence on elongation. The AT52-0.4Mn alloy had the highest elongation in theory, but it was not included in the nine designed kinds of alloys yet. AT52-0.2Mn alloy had the highest elongation in the nine alloys (28.4%).

## 1. Introduction

Magnesium alloys are widely used in the automotive field owing to their rich resources, low density, high specific strength, and specific stiffness [1,2,3,4,5,6]. However, magnesium alloys have the characteristics of poor deformability, poor corrosion resistance, and low strength compared with Al alloys, which seriously restrict the wide application [7,8,9]. Therefore, much effort has been devoted to study magnesium alloys with excellent comprehensive properties.

Deformation (extrusion, rolling, and so on) and alloying are important ways to obtain excellent comprehensive properties of magnesium alloys. Extrusion can markedly improve the strength by refining the grains [10], and the method has been used to improve the strength of commercial magnesium alloys, rare-earth magnesium alloys, and non-rare-earth magnesium alloys [11,12,13].

Fine-grained strengthening and precipitation strengthening are commonly used to strengthen magnesium alloys. The addition of rare-earth elements can refine the grain and form the second phase, and a small amount of rare earth (RE) addition can achieve good comprehensive mechanical properties [14,15,16,17]. The yield strength of Mg-11.7Gd-4.9Y-0.3Zr (wt.%) [18] reaches 500 MPa with its ultimate tensile strength (UTS) of 540 MPa. However, the high price of rare-earth elements significantly increases the cost of rare-earth magnesium alloys. This restricts the commercial application of magnesium alloys, so it is imminent to develop rare-earth free magnesium alloys [19,20].

Mg-Al alloys are the most common commercial magnesium alloys, such as AZ31, AZ80, and AM60. Al is mainly precipitated along a grain boundary in the form of Mg_17_Al_12_ phase. However, when the temperature exceeds 120 °C, the Mg_17_Al_12_ phase starts to soften. Then, the Mg_17_Al_12_ phase cannot play the role of a pinning-grain boundary in high temperatures, which leads to the reduction of mechanical properties [10,21]. In order to further expand the application of Mg-Al alloy, other alloy elements can be added to improve the properties.

The study of Mg-Al-Sn alloys is being widely concerned [21,22,23,24]. Sn is a very beneficial element for the mechanical properties of Mg alloys, and the Mg_2_Sn phase plays a significant role in fine-grain strengthening and precipitation strengthening [25,26,27,28,29]. In addition, research finds that [30,31] the doping of Sn can reduce the generalized stacking fault energy of basal slips and nonbasal slips of Mg, which benefits the activation of basal slip and nonbasal slips, and thus enhancing the ductility of Mg. Previous research has shown that the Mg-Al-Sn alloy possesses excellent comprehensive mechanical properties. J. She et al. [23] found that the yield strength of Mg-6Al-3Sn-0.3Mn (wt.%) reaches 226 MPa, with UTS of 326 MPa, and elongation (EL) to failure of 14.3%.

In addition, the Mn element also affects the mechanical properties of the Mg alloy. The content of Mn in magnesium alloys is usually less than 1.5% [32,33,34]. According to the Mg-Mn binary phase diagram, the ultimate solid solubility of Mn in magnesium is 3.4%. The addition of a minor Mn element to molten alloy can remove Fe and improve corrosion resistance [35,36,37]. The mechanism of Mn removing Fe from magnesium alloys is not yet clear. It is generally believed that Mn can form an MnFe mixture or an (Fe, Mn) Al_3_ phase with Fe [38,39]. Furthermore, Mn can react with Al to form an Al-Mn phase. An Al-Mn phase can effectively refine the grain of magnesium alloy, and enhance the strength of magnesium alloy through fine-grain strengthening.

The effect of different elements on the microstructure and properties can be studied by orthogonal experimental design. Orthogonal experimental design is an effective mathematical statistics method to analyze the factors of each experiment scientifically [40,41,42,43]. This method can be used to design, compare, and analyze the tests with a neat array of orthogonal tables, and to find the best combination of the level of each factor. In this experiment, the influence of three elements, Al, Sn, and Mn, on the properties of magnesium alloy was investigated by orthogonal experimental design.

## 2. Materials and Methods

The main indexes to evaluate the microstructures and mechanical properties difference of magnesium are grain size, texture intensity, UTS, tensile yield strength (TYS), and EL. The composition of the alloy is designed by the orthogonal method. According to previous research [1,23,24], the alloy is based on Mg-Al-Sn-Mn system. The content of Al is 5–7 wt.%, the content of Sn is 1–3 wt.%, and the content of Mn is 0–0.4 wt.%.

The materials investigated in the present work are prepared in the same way as our previous work [7]. Prior to extrusion, the heat-treated samples were preheated at 350 °C for 1 h in a resistance furnace, and then extruded at 350 °C with an extrusion ratio of 25:1.

Before the observation of the microstructure of the as-cast and extruded samples, the corresponding samples were etched with a mixture of 1 mL of acetic acid, 6 mL of anhydrous ethanol, 1 mL of distilled water, and an appropriate amount of picric acid. The microstructure of the alloy was observed using a metallographic microscope (MDS, OPTEC, Chongqing, China) and scanning electron microscopy (VEGA II LMH, TESCAN, Brno, Czech Republic, JSM-7800F, JEOL, Tokyo, Japan). The composition of the alloys and the second phase were identified using an Energy Dispersive Spectrometer (EDS, INCA Energy, Oxford Inc., Oxford, UK), and X-ray diffraction (XRD, D/max-1200, Rigaku, Tokyo, Japen). The size of the sample used for XRD is 15 mm in diameter and 5 mm in thickness. The samples were polished with sandpaper until 2000#, and then cleaned with alcohol. Specimens preparation for electron backscatter diffraction (EBSD) observation consisted of mechanical grinding and electropolishing in an AC2 solution, using a voltage of 20 V for 60 s under a controlled temperature of −20 °C. Finally, the samples were cleaned with ethanol. The EBSD data was collected using an JOEL-JSM7800F field emission scanning electron microscope (JSM-7800F, JEOL, Tokyo, Japan) equipped with an HKLEBSD system operating at 20 kV with a step size of 1.0 µm.

For mechanical characterization, tensile tests were carried out using a universal material machine (SANSI GMT5105) with a strain rate of 3 mm/min at room temperature. The tensile direction was parallel to the extrusion direction.

## 3. Results

### 3.1. Microstructures of As-Cast Mg-Al-Sn-Mn Magnesium Alloys

**Figure 1:**
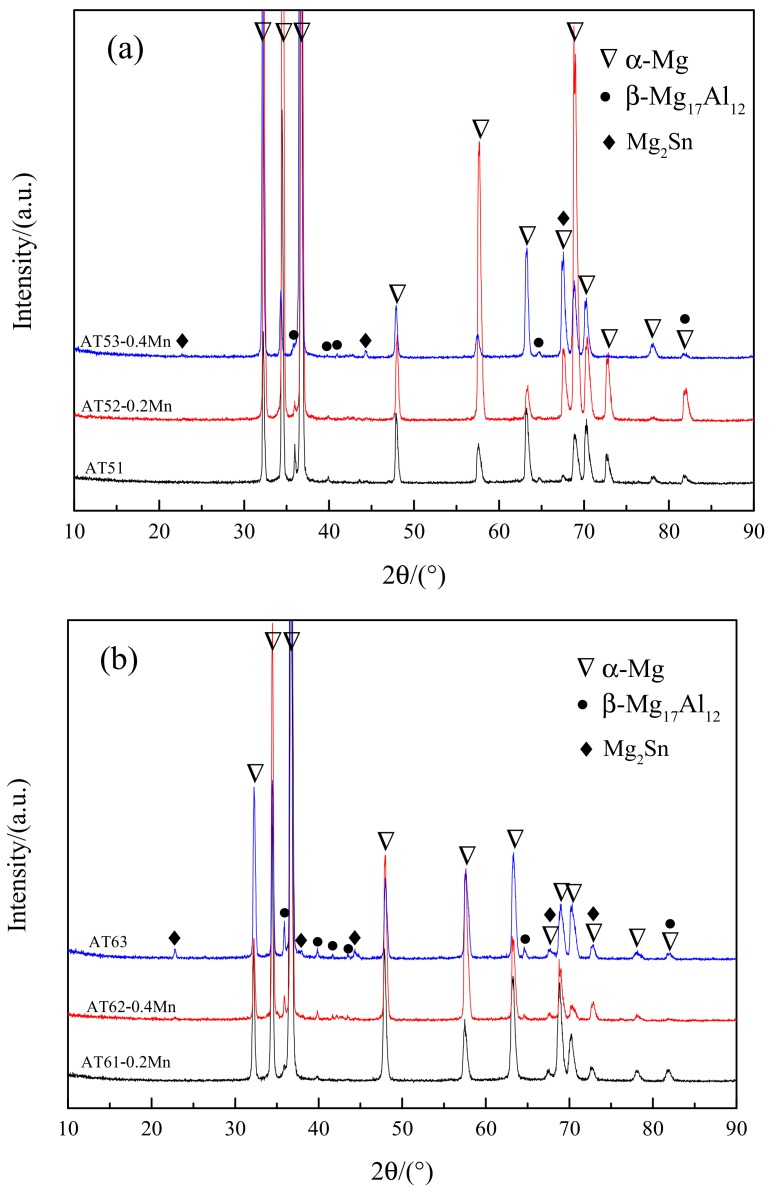

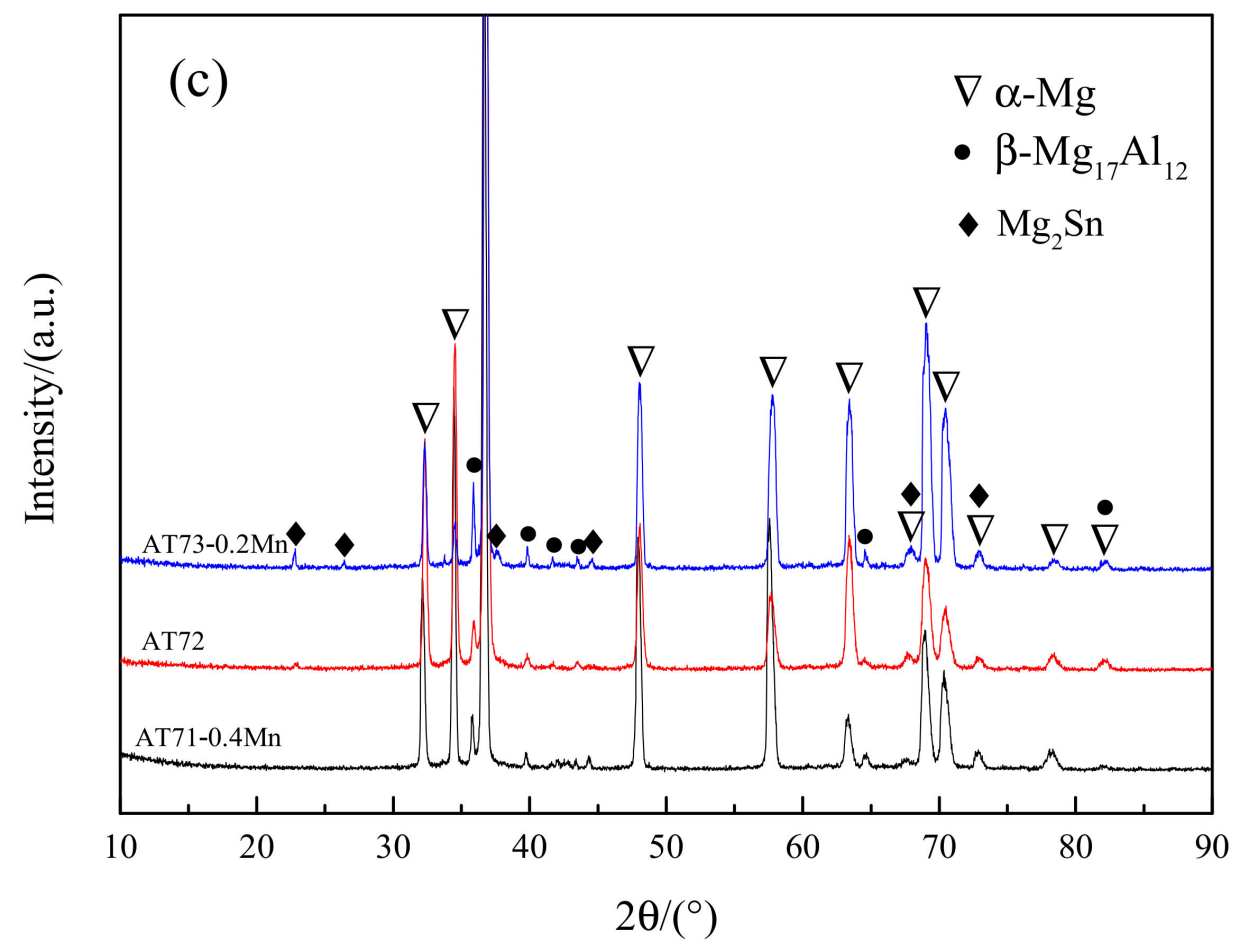
X-ray diffraction (XRD) results of as-cast Mg-Al-Sn-Mn alloys (**a**) AT51, AT52-0.2Mn, AT53-0.4Mn; (**b**) AT61-0.2Mn, AT62-0.4Mn, AT63; (**c**) AT71-0.4Mn, AT72, AT73-0.2Mn.

Figure 1 is the XRD pattern results of as-cast Mg-Al-Sn-Mn alloys; the common phases of nine alloys are α-Mg, β-Mg_17_Al_12_, and Mg_2_Sn.

The XRD pattern with the same content of Al in Figure 1 shows that the β-Mg_17_Al_12_ diffraction peak is more obvious with the increase of the content of Sn. When the content of Sn is 3 wt.%, the Mg_2_Sn diffraction peaks appear in AT53-0.4Mn, AT63, and AT73-0.2Mn alloys. This indicates that with the increase of Sn, the content of β-Mg_17_Al_12_ and Mg_2_Sn phase in the cast alloy increases.

When the content of Sn is constant, with the increase of Al, the β-Mg_17_Al_12_ diffraction peak becomes increasingly obvious, and the Mg_2_Sn diffraction peak becomes more obvious, too. As Al content increases, the solid solubility of Sn in Mg decreases, thus producing Mg_2_Sn phase.

**Figure 2:**
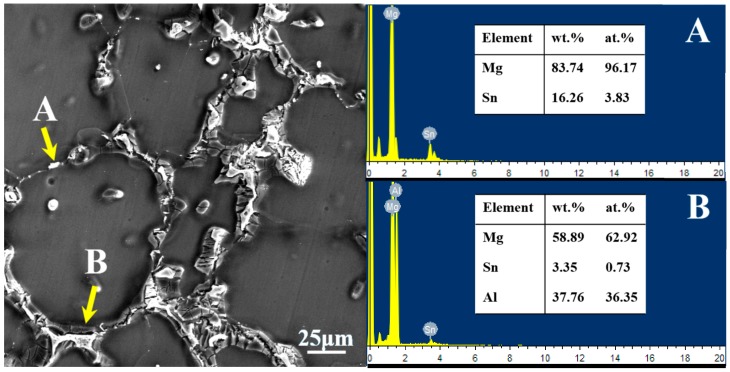
The Energy dispersive spectrometry (EDS) results of the main phase in the as-cast AT63 alloy. (**A**) Mg_2_Sn; (**B**) Mg_17_Al_12_.

SEM image and EDS results of the as-cast AT63 alloy are given in Figure 2. It can be found that the gray-white eutectic is α-Mg+β-Mg_17_Al_12_ (as shown in B in Figure 2). The small bright white strip and globular particles are Mg_2_Sn phase from the combination of EDS analysis and XRD results (as shown in Figure 1b and Figure 2A).

**Figure 3:**
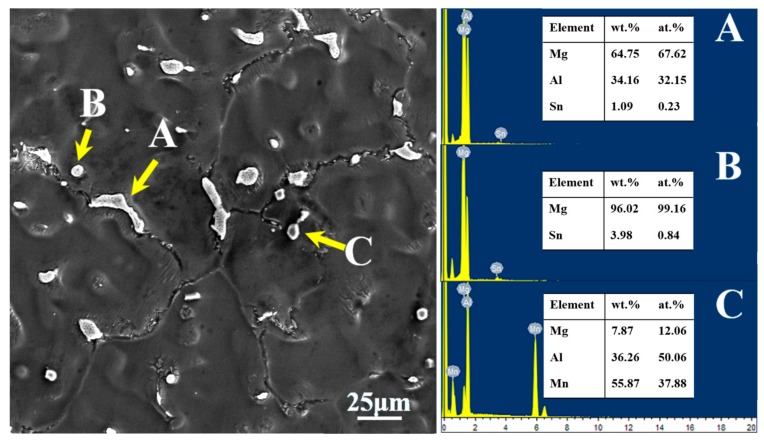
The EDS results of the main phases in the as-cast AT71-0.4Mn alloy. (**A**) Mg_17_Al_12_; (**B**) Mg_2_Sn; (**C**) Al-Mn phase.

SEM image and EDS results of the main phases in the as-cast AT71-0.4Mn alloy are presented in Figure 3. The combination of EDS analysis and XRD results shows that the long-stripe particles along the grain boundaries are the β-Mg_17_Al_12_ phase (as shown in Figure 3A), and the small spheroidal particles distributed along the grain boundaries or inside grains are Mg_2_Sn phase (as shown in Figure 3B). Besides, many irregular polygonal particles can also be observed in the as-cast AT71-0.4Mn alloy, these irregular polygonal particles are Al-Mn phase (as shown in Figure 3C).

### 3.2. Microstructure of Extruded Mg-Al-Sn-Mn Magnesium Alloys

**Figure 4:**
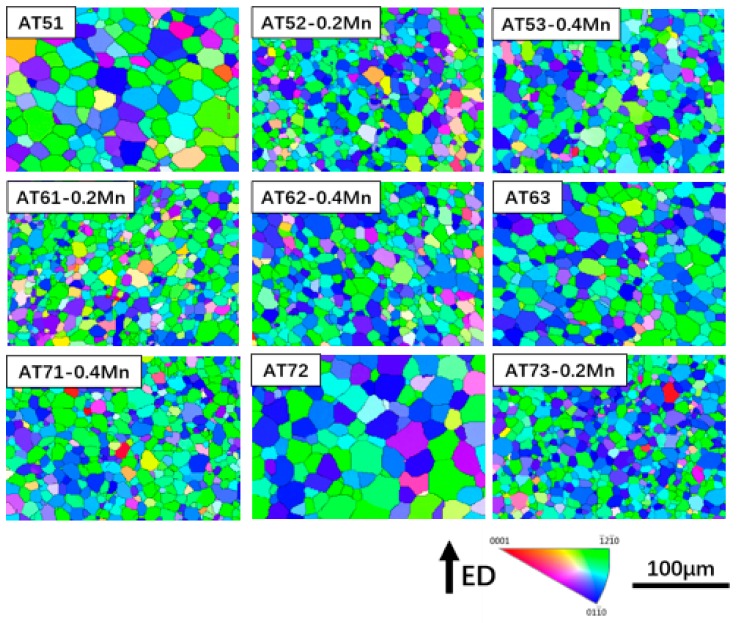
Electron backscatter diffraction (EBSD) inverse-pole figure maps of extruded Mg-Al-Sn-Mn magnesium alloys.

Figure 4 presents EBSD inverse-pole figure (IPF) maps of the extruded Mg-Al-Sn-Mn magnesium alloys. IPF maps of nine kinds of extruded alloys are arranged in the order of orthogonal experimental design, too.

Inspection of the images in Figure 4 reveals that dynamic recrystallization occurs in nine alloys during the hot-extrusion process. The cast dendrite structure changes into the recrystallized equiaxed grain.

The extreme-difference analysis method can easily and intuitively show the influence of each element on the alloy. Table 1 gives the orthogonal-test parameters and results analysis table under the grain-size index from EBSD results.

The R value of each element in Table 1 ranks in the order of R(C), R(B), and R(A). It shows that the content of Mn elements in the alloy has the greatest influence on the grain size, followed by Sn and Al. For magnesium alloys, Mn is the most significant grain-refining element [44,45]. The Mn and Al elements form the primary Al–Mn phase from the liquid [44]. The Al–Mn phase also has a high melting point (642 °C) that cannot be dissolved into the matrix during homogenization. In the process of hot extrusion, the Al–Mn phase acts as a heterogeneous nucleation point, which restricts the growth of the dynamic recrystallized grain [32,34,45]. In addition, the fine Mg_2_Sn phase precipitates formed during the extrusion process are distributed along the grain boundaries and within the grain interiors [1] (as shown in the following SEM results). Mg_2_Sn precipitates also play an important role in restricting the growth dynamic recrystallized (DRXed) grain via grain-boundary pinning, which is consistent with previously reported results for extruded TAZ711 [46] and TAZ811 [47] alloys. Therefore, Sn can also effectively refine the grain size of an Mg-Al-Sn-Mn alloy. Al elements are completely dissolved into the matrix during homogenization process. There is also no second-phase precipitate formed during extrusion. Therefore, the effect of Al on Mg-Al-Sn-Mn alloy grain size is the smallest.

**Figure 5:**
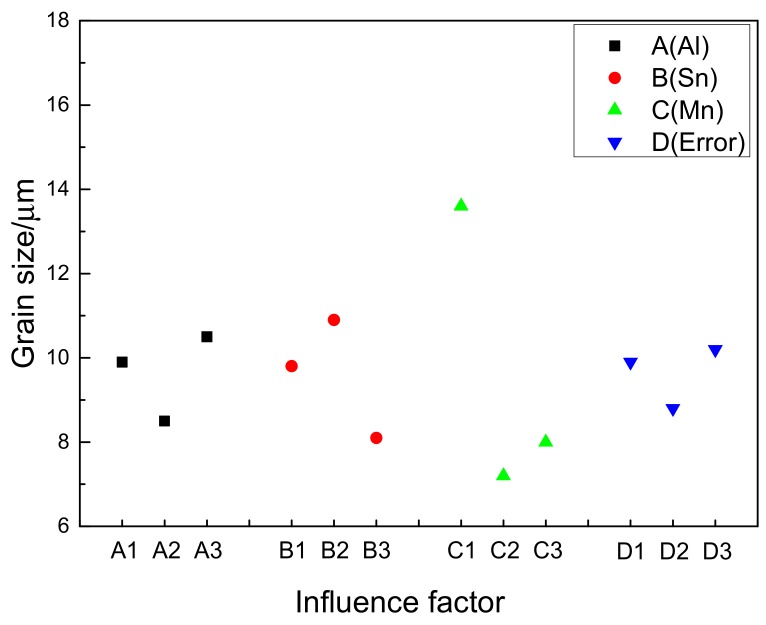
Factors and Index trends diagram of grain size for nine trial samples.

In order to understand the influence of Al, Sn, and Mn content on the grain size for the extruded Mg-Al-Sn-Mn magnesium alloys, the diagram of the relationship between factors and indicators (grain size) is shown in Figure 5. As shown in Figure 5, Mn is the main factor affecting grain size. With the content of Mn from 0 wt.% to 0.2 wt.%, the grain diameter decreases rapidly. However, when the content of Mn is increased to 0.4 wt.%, there is a rising trend. Therefore, the optimum Mn element content is 0.2 wt.%. Similarly, the best choice of Al and Sn are 6 wt.% and 3 wt.%, respectively. So, the AT63-0.2Mn alloy has the minimum grain size, in theory. It is not included in the designed nine kinds of alloys, yet. The AT61-0.2Mn and AT73-0.2Mn alloys have the smallest grain size in all alloys, reaching 6.8 μm.

**Figure 6:**
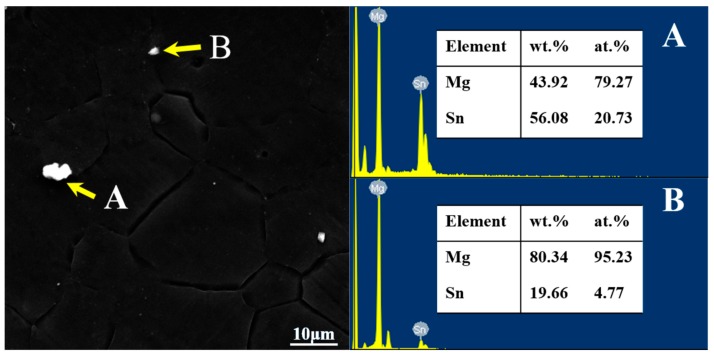
The scanning electron microscopy (SEM) and EDS results of the main phase in the as-extruded AT63 alloy. (**A**) and (**B**) are Mg_2_Sn with different sizes.

**Figure 7:**
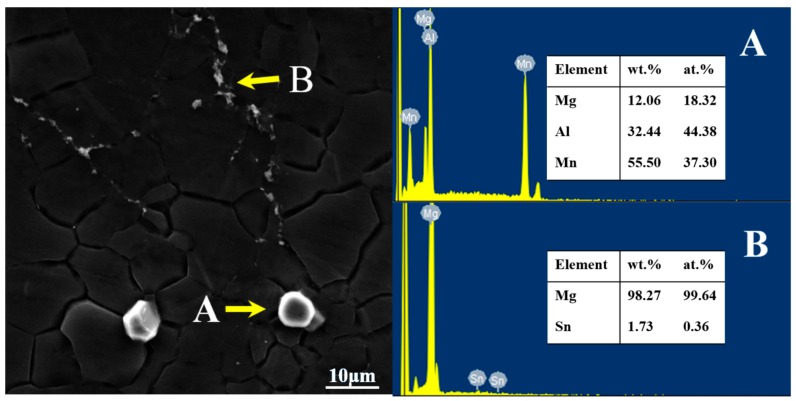
The EDS results of the main phase in the as-extruded AT71-0.4Mn alloy. (**A**) Al-Mn phase; (**B**) Mg_2_Sn.

In order to determine the type, morphology and distribution of the second phases in extruded Mg-Al-Sn-Mn magnesium alloys, the SEM and EDS of the extruded AT63 alloy and AT71-0.4Mn alloy were carried out. The results are shown in Figure 6 and Figure 7.

As can be seen from Figure 6, there are bright white particles in the extruded AT63 alloy with different sizes (as shown in A and B). The EDS analysis shows that the bright white particles are the Mg_2_Sn phase. The β-Mg_17_Al_12_ phase in the cast alloy was completely dissolved into the magnesium matrix during the homogenization process, and it was not precipitated during the extrusion process.

From Figure 7, there are several lumpy white particles and continuous white particles along the grain boundary in the extruded AT71-0.4Mn alloy. The lumpy white second phase in the alloy is mainly the enrichment area of Al and Mn elements, indicating that the lumpy white particle in the alloy (as shown in Figure 7A) is the second Al-Mn phase. The Sn element is almost evenly distributed on the Mg matrix, indicating that most of the Sn elements in Mg-Al-Sn-Mn magnesium alloy are dissolved into the magnesium matrix. Only a part of Sn and Mg form the Mg_2_Sn phase on the grain boundary (as shown in Figure 7B). Similar to the condition of AT63, the Mg_17_Al_12_ phase in the extruded AT71-0.4Mn alloy has not been detected, either.

### 3.3. Textures of Extruded Mg-Al-Sn-Mn Magnesium Alloy

**Figure 8:**
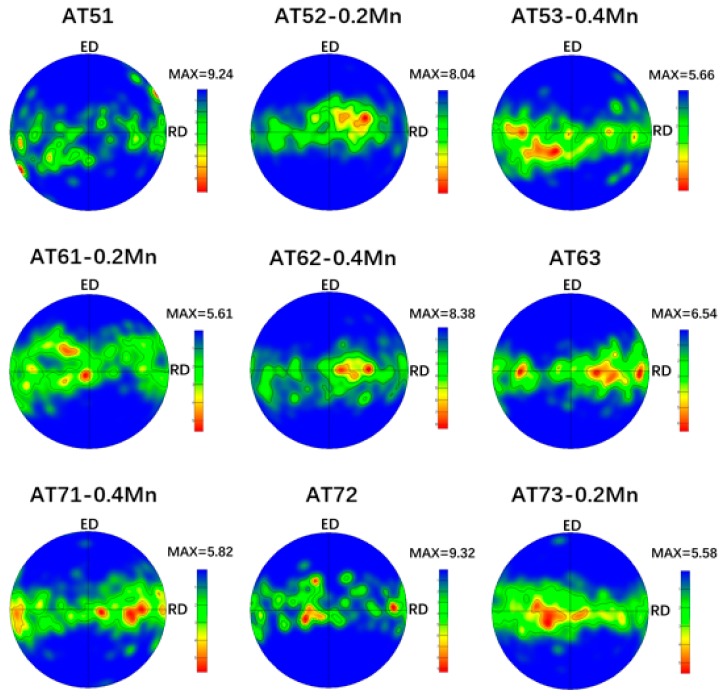
(0001) pole figures of extruded Mg-Al-Sn-Mn magnesium alloys.

Figure 8 shows the (0001) pole figures of extruded Mg-Al-Sn-Mn magnesium alloys; the as-extruded alloys exhibit a typical extruding texture with (0001) basal planes parallel to the extrusion direction (ED), which is a typical fiber texture in extruded magnesium alloys [36,48]. In addition, the maximum texture intensity varies with element content. The orthogonal test parameters and results analysis are shown in Table 2.

It can be obtained from Table 2 that R value of each element ranks in the order of R(B), R(C), and R(A). It shows that Sn has the greatest influence on the maximum texture intensity, followed by Mn, and the content of Al has the smallest effect. The maximum intensity of texture is often related to the DRX region. It has been reported that the Mg-4.5Zn-1.1Ca alloy extruded at lower temperatures exhibited a stronger texture due to the lower fraction of the DRXed region [49]. In the present work, the fractions of unDRXed grains are quite low, and it cannot be considered as a reason explaining the texture intensity change.

In addition, the strength of texture is related to the content of second phase and grain size. The increase of the second phase and the decrease of grain size will weaken the texture intensity [50,51]. On the one hand, the presence of Mg_2_Sn precipitates reduces the rate of tensile twinning due to the pinning of twin boundary [52]. On the other hand, the addition of Sn obviously affects grain size. Hence, the Sn element has the greatest influence on the texture intensity. The addition of Mn will obviously reduce the grain size and weaken the texture intensity. Al cannot form second-phase particles on a hot extruded process and has little effect on grain size (from Figure 2). So, Al content is the smallest factor affecting texture intensity.

**Figure 9:**
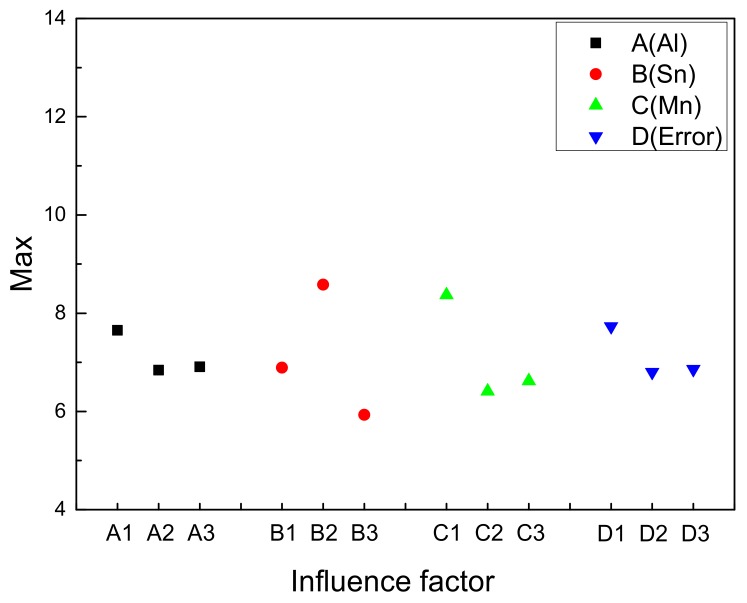
Factors and Index trends diagram of maximum texture intensity for nine trial samples.

Figure 9 shows the Factors and Index trends diagram of maximum texture intensity for nine trial samples. It can be used to understand the influence of Al, Sn, and Mn content on maximum texture intensity for extruded Mg-Al-Sn-Mn magnesium alloys. The change of grain size is the main factor affecting the texture intensity of magnesium alloy. Borkar et al. [53] revealed that the fine grains have relatively more random orientations than medium-to-large-sized grains; thus, a weaker texture happens. Therefore, the influence of element content on texture intensity in Figure 9 is similar to that of grain size in Figure 5. So, the AT63-0.2Mn alloy has the maximum texture intensity. It is not yet included in the designed nine kinds of alloys. However, there was little change in Al content of 6 wt.% and 7 wt.%. The AT73-0.2Mn alloy has the smallest maximum texture intensity in all alloys.

### 3.4. Mechanical Properties of Extruded Mg-Al-Sn-Mn Magnesium Alloys at Room Temperature

**Figure 10:**
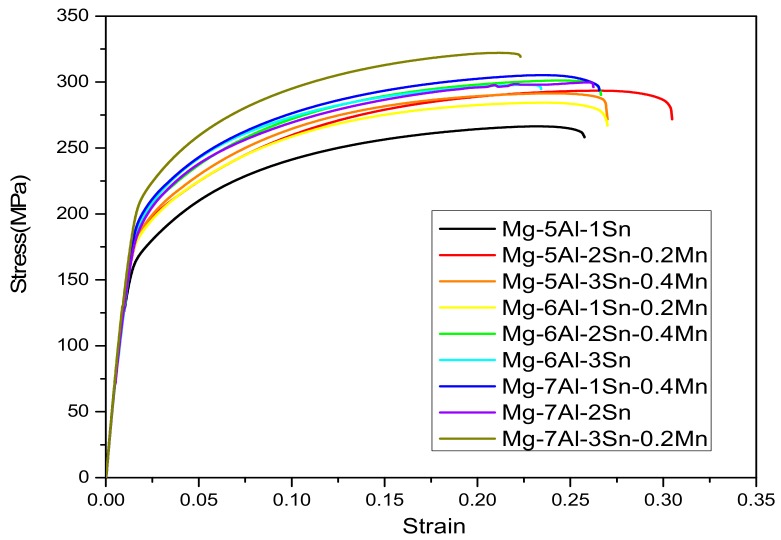
The stress-strain curves of the extruded Mg-Al-Sn-Mn alloy.

Figure 10 displays the stress-strain curvers of the extruded Mg-Al-Sn-Mn alloys. Table 3 gives the corresponding mechanical properties, listed in terms of TYS, UTS, and EL. It can be seen that the UTS of Mg-7Al-3Sn-0.2Mn is the highest and the UTS is up to 322 MPa, and the EL of Mg-5Al-2Sn-0.2Mn is the highest, reaching 28.4%.

Table 4 is the orthogonal test parameters and results analysis table under the UTS index. The R value of each element in Table 4 ranks in the order of R(A), R(B), and R(C). It shows that the content of Al has the greatest influence on the UTS of the alloy, followed by the content of Sn, and the content of Mn has the least influence. Al element improves UTS of magnesium alloy by solid solution strengthening owing to no Mg–Al phase is formed. This is the main strengthening mechanism of Mg-Al-Sn-Mn alloy. Thus, Al has the greatest influence on UTS. For Sn element, the Mg_2_Sn second phase can improve the UTS by precipitation strengthening and fine-grained strengthening mechanism. For Mn, as the content of Al-Mn phase is very low, UTS is mainly improved by fine-grained strengthening mechanism. Therefore, the effect of Mn on Mg-Al-Sn-Mn alloy UTS is the smallest.

**Figure 11:**
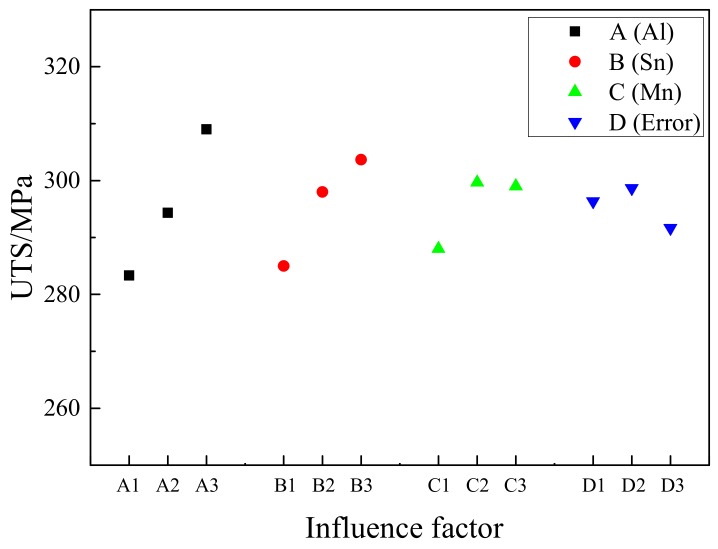
Factors and Index trends diagram of UTS for nine trial samples.

In order to understand the influence of Al, Sn and Mn content on UTS of the extruded Mg-Al-Sn-Mn magnesium alloy, the diagram of the relationship between factors and indicators (UTS) is shown in Figure 11.

From Figure 11, it is concluded that Al is the main factor affecting the UTS of the alloy. With the Al content from 5 wt.% to 7 wt.%, the UTS of the alloy almost increases linearly, so the best Al content is 7 wt.%. As the secondary factor, the content of Sn, from 1 wt.% to 3 wt.%, also has obvious strength enhancement, and the optimum content of Sn is 3 wt.%. As for Mn content from 0 wt.% to 0.2 wt.%, the UTS of the alloy is obviously enhanced. But when the content of Mn reaches 0.4 wt.%, the strength of the alloy decreases. So, the optimum content of Mn was 0.2 wt.%.

Above all, the optimum composition of the alloy with the largest UTS is Mg-7 Al-3Sn-0.2Mn (AT73-0.2Mn). The UTS of the best composite alloy is the highest in this test sample, reaching 322 MPa.

**Figure 12:**
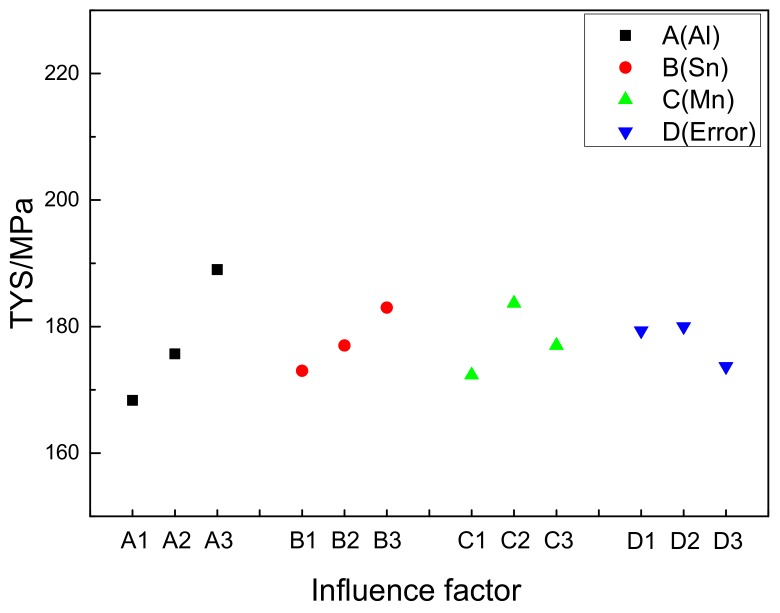
Factors and Index trends diagram of tensile yield strength for nine trial samples.

Table 5 is the orthogonal test parameters and results analysis table under the TYS index. Figure 12 is the Factors and Index trends diagram of TYS for nine trial samples. The influence of Al, Sn, and Mn content on TYS is almost the same as that of UTS. But the effect of grain size on TYS is more obvious than UTS. So, The R value of each element in Table 5 ranks in the order of R(A), R(C), and R(B). It shows that the content of Al elements in the alloy has the greatest influence. And the effect of Sn content on TYS is more obvious than that of Mn. From Figure 12, it can be seen that the Mg-7Al-3Sn-0.2Mn (AT73-0.2Mn) Mg alloy has the largest TYS (202 MPa). It is same to the UTS results.

Similar to the analysis of the strength index, it can be concluded from Table 6 that the R value ranks in the order of R(B), R(A), and R(C). The Sn content has the greatest influence on the elongation, and the R value is 3.47. The second is Al, and the content of Mn has the smallest effect on the elongation. The reason is that the addition of Sn to Mg alloys can decrease the stacking fault energy of the alloys, resulting in the improved activity of basal slip system {0001} <11$\bar{2}$0> and the activation of the {10$\bar{1}$1} <11$\bar{2}$0> and {11$\bar{2}$2} <11$\bar{2}$3> slip systems [30]. So, the addition of Sn makes the elongation increase. However, when excessive Sn is added, Mg_2_Sn hinders the movement of dislocation, resulting in the increase of the strength and decrease of the elongation. The Al element is completely dissolved into the matrix, resulting in lattice distortion. The increase of the Al content leads to the increase of lattice distortion. Then, it leads to the increase of the strength and decrease of elongation. The addition of Mn can effectively refine the grain size, leading to strength and elongation increasing at the same time.

**Figure 13:**
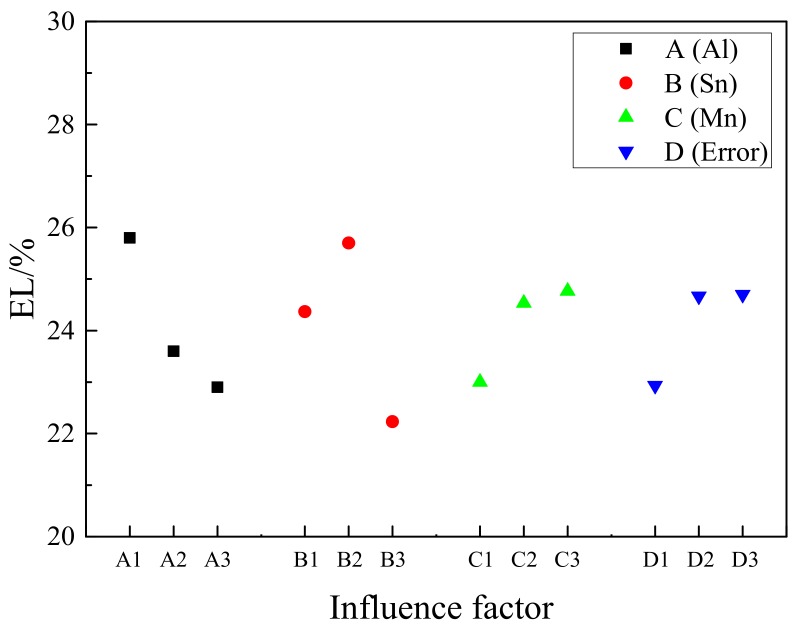
Factors and Index trends diagram of elongation for nine trial samples.

Combining with Figure 13, when the content of Al increases from 5 wt.% to 7 wt.%, the elongation of the alloy decreases and the optimum Al content is 5 wt.%. It is contrary to the trend of strength. The elongation increased greatly when the Sn addition increased from 1 wt.% to 2 wt.%. However, when the Sn content increased to 3 wt.%, the elongation decreased significantly. So, the optimum selection of Sn content is 2 wt.%. Similar to the Al and Sn selection methods, the optimum content of Mn is 0.4 wt.%.

Therefore, in theory, the AT52-0.4 Mn has the highest elongation, but it is not in the nine alloys of this design. As the content of Mn has little effect on the elongation, the content of Mn can be selected to 0.2 wt.%.

Above all, the optimum composition of the alloy with the highest elongation is Mg-5Al-2Sn-0.2Mn (AT52-0.2Mn). The EL of the best composite alloy is the highest in this test sample, reaching 28.4%.

## 4. Conclusions

In summary, this study is set out to investigate the effect of Al, Sn, and Mn elements on the grain size, texture intensity, strength, and elongation. Through orthogonal experimental design, this study has identified the influence of these three elements under different index. The following conclusions can be made from this study:

1. The influence of three alloy elements on the grain size is in order of Mn, Sn, Al. The AT63-0.2Mn alloy has the minimum grain size in theory. It is not included in the designed nine kinds of alloys yet. The AT61-0.2Mn and AT73-0.2Mn alloys have the smallest grain size in the nine alloys (6.8 μm).
2. The influence of three alloy elements on the maximum texture intensity is in the order of Sn, Mn, Al. The AT63-0.2Mn alloy has the minimum maximum texture intensity in theory. It is not included in the designed nine kinds of alloys, yet. AT61-0.2Mn alloy has the smallest maximum texture intensity in the 9 alloys (5.58).
3. The influence of three alloy elements on the UTS and TYS are in the order of Al, Sn, Mn and Al, Mn, Sn, respectively. The AT73-0.2Mn alloy has the maximum UTS and TYS in theory. Its UTS and TYS are 322 MPa and 202 MPa, respectively.
4. The influence of three alloy elements on the elongation is in the order of Sn, Al, Mn. The AT52-0.4Mn alloy has the highest elongation in theory. It is not included in the designed nine kinds of alloys yet. AT52-0.2Mn alloy has the highest elongation in the nine alloys (28.4%).

## Figures and Tables

**Table 1:** Orthogonal test parameters and results analysis table under the grain size index.

Number	Factor				Index
	Al (A)	Sn (B)	Mn (C)	Null (D)	Grain Size (μm)
1(AT51)	1	1	1	1	14.4 ± 2.5
2(AT52-0.2Mn)	1	2	2	2	8.0 ± 1.6
3(AT53-0.4Mn)	1	3	3	3	7.4 ± 1.3
4(AT61-0.2Mn)	2	1	2	3	6.8 ± 1.2
5(AT62-0.4Mn)	2	2	3	1	8.4 ± 1.5
6(AT63)	2	3	1	2	10.2 ± 1.8
7(AT71-0.4Mn)	3	1	3	2	8.3 ± 1.5
8(AT72)	3	2	1	3	16.3 ± 2.6
9(AT73-0.4Mn)	3	3	2	1	6.8 ± 1.2
K1	29.8	29.5	40.9	29.6	
K2	25.4	32.7	21.6	26.5	
K3	31.4	24.4	24.1	30.5	
R	2	2.77	6.43	1.33	

**Table 2:** Orthogonal test parameters and results analysis table under the maximum texture intensity index.

Number	Factor				Index
	Al (A)	Sn (B)	Mn (C)	Null (D)	Max Intensity
1(AT51)	1	1	1	1	9.24
2(AT52-0.2Mn)	1	2	2	2	8.04
3(AT53-0.4Mn)	1	3	3	3	5.66
4(AT61-0.2Mn)	2	1	2	3	5.61
5(AT62-0.4Mn)	2	2	3	1	8.38
6(AT63)	2	3	1	2	6.54
7(AT71-0.4Mn)	3	1	3	2	5.82
8(AT72)	3	2	1	3	9.32
9(AT73-0.4Mn)	3	3	2	1	5.58
K1	22.94	20.67	25.10	23.20	
K2	20.53	25.74	19.23	20.40	
K3	20.72	17.78	19.86	20.59	
R	0.80	2.65	1.96	0.93	

**Table 3:** The result of the tensile test at room temperature of the extruded Mg-Al-Sn-Mn alloy.

Number	Alloy	YS/MPa	UTS/MPa	EL%
1	AT51	160 ± 4	266 ± 4	23.8 ± 1.6
2	AT52-0.2Mn	176 ± 2	293 ± 3	28.4 ± 2.0
3	AT53-0.4Mn	169 ± 2	291 ± 1	25.2 ± 1.8
4	AT61-0.2Mn	173 ± 3	284 ± 4	24.9 ± 1.3
5	AT62-0.4Mn	176 ± 5	301 ± 2	24.7 ± 1.2
6	AT63	178 ± 4	298 ± 2	21.2 ± 1.5
7	AT71-0.4Mn	186 ± 2	305 ± 3	24.4 ± 1.2
8	AT72	179 ± 1	300 ± 2	24.0 ± 0.9
9	AT73-0.2Mn	202 ± 3	322 ± 3	20.3 ± 1.3

**Table 4:** Orthogonal test parameters and results analysis table under the ultimate tensile strength (UTS) index.

Number	Factor				Index
	A (Al)	B (Sn)	C (Mn)	D (null)	UTS/MPa
1(AT51)	1	1	1	1	266
2(AT52-0.2Mn)	1	2	2	2	293
3(AT53-0.4Mn)	1	3	3	3	291
4(AT61-0.2Mn)	2	1	2	3	284
5(AT62-0.4Mn)	2	2	3	1	301
6(AT63)	2	3	1	2	298
7(AT71-0.4Mn)	3	1	3	2	305
8(AT72)	3	2	1	3	300
9(AT73-0.4Mn)	3	3	2	1	322
K1	850	855	864	889	
K2	883	894	899	896	
K3	927	911	897	875	
R	25.7	18.7	11.7	7	

**Table 5:** Orthogonal test parameters and results analysis table under the tensile yield strength (TYS) index.

Number	Factor				Index
	A (Al)	B (Sn)	C (Mn)	D (null)	TYS/MPa
1(AT51)	1	1	1	1	160
2(AT52-0.2Mn)	1	2	2	2	176
3(AT53-0.4Mn)	1	3	3	3	169
4(AT61-0.2Mn)	2	1	2	3	173
5(AT62-0.4Mn)	2	2	3	1	176
6(AT63)	2	3	1	2	178
7(AT71-0.4Mn)	3	1	3	2	186
8(AT72)	3	2	1	3	179
9(AT73-0.4Mn)	3	3	2	1	202
K1	505	519	517	538	
K2	527	531	551	540	
K3	567	549	531	521	
R	20.7	10.0	11.3	6.3	

**Table 6:** Orthogonal test parameters and results analysis table under the elongation (EL) index.

Number	Factor				Index
	A (Al)	B (Sn)	C (Mn)	D (null)	El/%
1(AT51)	1	1	1	1	23.8
2(AT52-0.2Mn)	1	2	2	2	28.4
3(AT53-0.4Mn)	1	3	3	3	25.2
4(AT61-0.2Mn)	2	1	2	3	24.9
5(AT62-0.4Mn)	2	2	3	1	24.7
6(AT63)	2	3	1	2	21.2
7(AT71-0.4Mn)	3	1	3	2	24.4
8(AT72)	3	2	1	3	24.0
9(AT73-0.4Mn)	3	3	2	1	20.3
K1	77.4	73.1	69.0	68.8	
K2	70.8	77.1	73.6	74.0	
K3	68.7	66.7	74.3	74.1	
R	2.90	3.47	1.77	1.77	
